# Drivers of *Perkinsus marinus* and *Haplosporidium nelsoni* prevalence and intensity in oyster reefs around Sapelo Island, Georgia

**DOI:** 10.1017/S0031182025101431

**Published:** 2026-02

**Authors:** Wil Atencio, Shelby Ziegler, Stephen Greiman, John Carroll

**Affiliations:** 1 Department of Biology, Georgia Southern University, Statesboro, GA, USA; 2 Department of Environmental Sciences, University of Virginia, Charlottesville, VA, USA; 3 Department of Biology and Center for Biodiversity and Ecosystem Stewardship, Villanova University, Villanova, PA, USA

**Keywords:** eastern oyster, Haplosporidium, lag effects, Perkinsus, water quality

## Abstract

Parasites can strongly influence host populations, particularly when the host is an ecosystem engineer. Oysters are ecosystem engineers that support estuarine communities and fisheries but are susceptible to 2 protozoan parasites, *Perkinsus marinus* (causing Dermo) and *Haplosporidium nelsoni* (causing MSX). Although both parasites are known to be influenced by environmental conditions, fine-scale temporal and spatial patterns remain underexplored in southeastern US estuaries. We examined parasite prevalence and intensity biweekly from April to October 2023 across 4 intertidal reefs on Sapelo Island, Georgia, and analysed concurrent water quality data (temperature, salinity, dissolved oxygen, pH) to identify potential environmental drivers of parasite prevalence and intensity. Parasite prevalence was high overall, 88% of oysters were infected with at least 1 parasite, and 34% were co-infected. *Haplosporidium nelsoni* prevalence was consistently high across sites, while *P. marinus* prevalence showed greater spatiotemporal variability, increasing through late summer and fall. Models indicated a time-lagged effect of environmental conditions on *P. marinus* prevalence, specifically with temperature and dissolved oxygen. Prevalence of *H. nelsoni* remained high throughout the year among sites and was best explained by temperature variability, salinity, and dissolved oxygen. Intensity levels did not differ among sites for either parasite. Our results demonstrate that even at small spatial scales and over time, oyster–parasite dynamics are shaped by multiple, interacting environmental factors, with time-lagged responses particularly evident for *P. marinus*. Understanding these dynamics is essential for predicting disease impacts under changing environmental conditions and informing management, restoration, and aquaculture strategies.

## Graphical abstract

**Figure:**
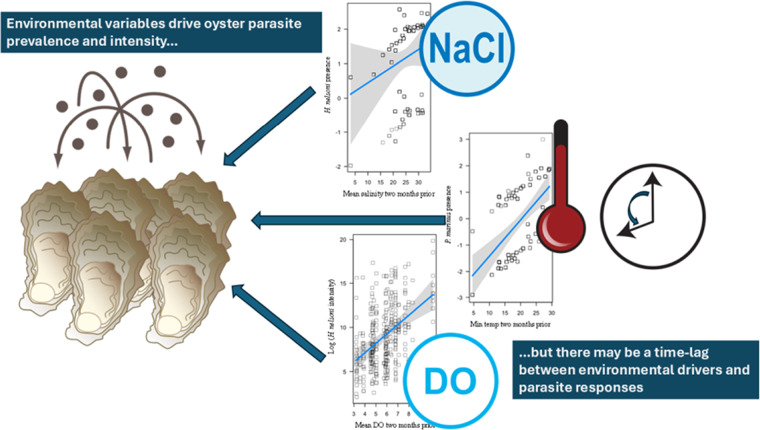

## Introduction

Parasites and pathogens are diverse and ubiquitous members of ecological communities that negatively impact host fitness (Lafferty and Kuris, 1999; Khan et al. 2019), including reduced host survival, reproduction, and growth, as well as changes in host behaviour (Combes, 1996; Poulin, 2013; Malek and Breitburg, 2016). The effects of parasites on their hosts can be both modulated and exacerbated by environmental conditions (Lafferty and Kuris, 1999), leading to variation of prevalence, intensity, and impact across time and space (Byers et al. 2008; Ziegler et al. 2024). Local environmental conditions and biotic interactions can influence host–parasite dynamics and the risk of infection and parasite prevalence (Byers et al. 2008). However, local-scale drivers may be nested within larger regional patterns, such as seasons, temperature, differential dispersal patterns of vectors, or the molecular basis of the parasite–host interaction (Brown et al. 1995; Bushek and Allen, 1996; Farnsworth et al. 2006; Byers et al. 2008). To understand how parasites affect individual hosts and regulate the dynamics of entire communities, it is critical to investigate biotic and abiotic drivers as well as the variability of parasite prevalence and intensity across spatiotemporal scales.

The relationship between hosts, parasites, and their environment is complex (Malek and Byers, 2017), particularly when the hosts are ecosystem engineers (Lenihan et al. 1999; Malek and Breitburg, 2016; Malek and Byers, 2017; Batchelor et al. 2023; Ziegler et al. 2024), which provide physical structure and resources and modify abiotic conditions for other organisms (Jones et al. 1997; Coen et al. 2007). Increases in parasite prevalence and intensity in ecosystem engineers can therefore cascade to affect the entire community (Malek and Breitburg, 2016; Ziegler et al. 2024). For example, increased disease prevalence in *Acropora* corals in the Indian Ocean driven by environmental stressors has led to reduced habitat complexity and biodiversity, causing an overall degradation of the reef community (Harvell et al. 1999, 2002; Bruno and Selig, 2007; Sharma and Ravindran, 2020). Understanding how multiple environmental factors interact to influence host–parasite dynamics of ecosystem engineers is critical because infectious diseases are among many threats that have led to their global decline (Harvell et al. 1999).

The eastern oyster, *Crassostrea virginica,* is a reef-building ecosystem engineer in coastal and estuarine environments ranging from New Brunswick, Canada to the Yucatan Peninsula, Mexico (Burford et al. 2014). In addition to being a commercially valuable food product, oysters play an essential ecological role by creating complex habitats for numerous other species, improving water quality, and stabilizing shorelines (Grabowski and Peterson, 2007; Ehrich and Harris, 2015; Walles et al. 2016; Watts et al. 2018). A combination of factors, including overharvest, pollution, habitat loss and degradation, climate change and disease, have caused drastic reductions of up to 85% of global oyster populations (Rothschild et al. 1994; Lotze et al. 2006; Worm et al. 2006; Beck et al. 2011; Zu Ermgassen et al. 2012; Bersoza Hernández et al. 2018), with subsequent consequences for coastal communities, leading to declines in fisheries and ecosystem function (Grabowski and Peterson, 2007; Beck et al. 2011). Due to the economic and ecological importance of oysters, considerable investments have been allocated to oyster aquaculture, management and restoration. The success of management efforts has faced many challenges, including parasites (Andrews, 1988; Lewis et al. 1992; Ewart and Ford, 1993).

Eastern oysters are susceptible to several parasites, including *Perkinsus marinus*, which causes Dermo disease, and *Haplosporidium nelsoni*, which causes MSX disease. Both parasites lead to reduced growth and reproduction, and eventually death (Burreson and Ragone Calvo, 1996; Ford and Tripp, 1996; Malek and Byers, 2017), and both have been implicated in mass mortality events throughout the range of eastern oysters (Andrews, 1988; Burreson, 1994; Burreson and Ragone Calvo, 1996; Carnegie and Burreson, 2012). However, the mechanisms for infection and spread are different across the 2 protozoans. Transmission of *P. marinus* is related to host density; the infective stages enter the water column through faeces and pseudofaeces from live, infected oysters and are subsequently directly ingested by oysters during filter feeding. (Ford and Tripp, 1996; Bushek et al. 2002; Malek and Byers, 2017). Once inside the oyster, *P. marinus* proliferates within haemocytes, eventually killing the haemocyte and releasing *P. marinus* cells that spread into various tissues until the infection becomes systemic (Burreson and Ragone Calvo, 1996; Bushek and Allen, 1996). Unlike *P. marinus,* the spread of *H. nelsoni* is independent of oyster density, fairly uniform over large areas of high salinity portions of estuaries (Ford and Tripp, 1996) and likely involves an intermediate host (Burreson, 1994). Within the oyster, *H. nelsoni* forms multinucleated plasmodia that can occur in the connective tissue or epithelium of all oyster tissue (Ford and Tripp, 1996; Carnegie and Burreson, 2012).

Given the negative impacts of both protozoan parasites, the roles that temperature and salinity play in diseases caused by *P. marinus* (Dermo) and *H. nelsoni* (MSX) have been extensively investigated (Haskin and Ford, 1982; Soniat, 1985; Paynter and Burreson, 1991; Ewart and Ford, 1993). Rapid proliferation occurs in waters >20°C (Guo and Ford, 2016), and mortality events are usually tied to high parasite prevalence coupled with high temperatures. Additionally, high salinity (>15 ppt) – typically increasing throughout spring and summer months in the southeastern USA – drives the proliferation and abundance of *P. marinus* and *H. nelsoni* (Ford and Haskin, 1982; Paynter and Burreson, 1991). Heightened impacts and range expansion of diseases may be caused by rising temperatures associated with climate change and dry conditions that produce increasing salinity in estuaries (Cook et al. 1998; Harvell et al. 1999; Guo and Ford, 2016; Carnegie et al. 2021). Other environmental factors such as low dissolved oxygen (DO) can decrease oyster immune response and allow for the progression of parasite infections (Breitburg et al. 2015). Low DO or hypoxia-related immunological suppression decreases oyster tissue pH as a result of a buildup of CO_2_, benefiting parasite proliferation and increasing the severity of disease (Paynter, 1996; Lenihan et al. 1999).

Though studies have investigated how multiple environmental factors drive Dermo and MSX disease dynamics (Lenihan et al. 1999; Batchelor et al. 2023; Ziegler et al. 2024), knowledge gaps remain regarding how water quality parameters synergistically influence the prevalence and intensity of *P. marinus* and *H. nelsoni* across a small spatial scale and over time. Therefore, we sought to determine the combined effects of temperature, salinity, DO and pH on the disease dynamics of these 2 protozoan parasites infecting oysters across sites in close spatial proximity, but that experience variable water quality conditions. Specifically, we examined patterns of prevalence and intensity for both parasites at 4 sites on Sapelo Island, Georgia, USA and assessed which combination of water quality parameters were most influential in driving the patterns of infection prevalence and intensity. We predicted that prevalence and intensity of both parasites would follow a seasonal pattern, peaking when water temperature and salinity was highest and that parasite prevalence and intensity would increase as DO and pH decrease.

## Materials and methods

### Study location and sampling

**Figure 1.**
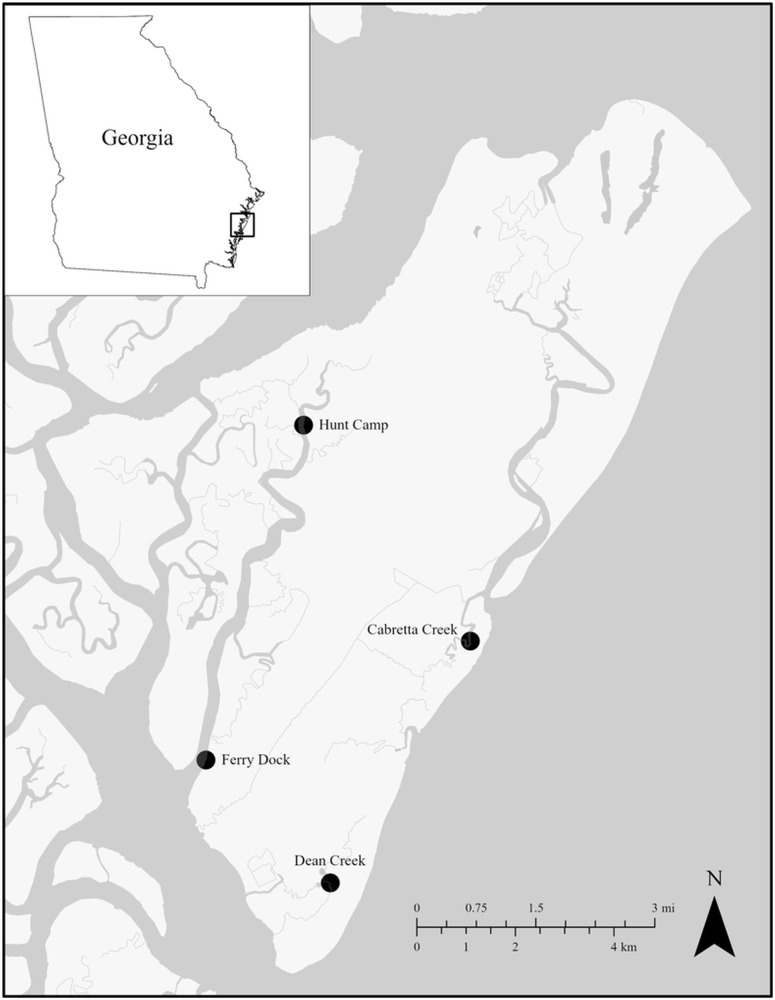
SINERR SWMP sites where oyster samples were collected from April through October 2023.

In order to explore how both *P. marinus* and *H. nelsoni* parasites are driven by local water quality conditions, we collected oysters from 4 sites around the Sapelo Island National Estuarine Research Reserve (SINERR) that have continuous water quality monitoring data as part of the National Estuarine Research Reserve (NERR) System-Wide Monitoring Program (SWMP): Hunt Camp (31°28’43”N 81°16’22”W), Cabretta Creek (31°26’19”N 81°14’19”W), Dean Creek (31°23’41”N 81°16’11”W) and Ferry Dock (31°25’04”N 81°17’46”W) (Figure 1). At biweekly intervals from April through October 2023, oysters were haphazardly sampled at each site. Initially, we excavated all live oysters from a single 0.25 m × 0.25 m quadrat for the first 3 collections. However, to reduce impacts of continued sampling on the same reefs, starting in June, we haphazardly collected 3–4 oyster culms per collection. For all sampling, oysters were returned to the lab and frozen until processing. In the lab, individual oysters were separated and cleaned, measured for left valve lengths (LVL) and width (in mm), weighed, and dissected. A small piece of gill tissue sample (>150 mg) was excised from 10 randomly selected oysters, preserved in 95% ethanol and stored at −20°C until later DNA extraction (see Table S1 for total numbers of oysters sampled).

Genomic DNA was extracted from oyster gill tissue using an Omega E.Z.N.A® Mollusc and Insect Extraction Kit following the protocol provided by the manufacturer (Omega Bio-tek, Inc., Norcross, GA, USA). Extracted DNA was quantified using a NanoDrop^TM^ 2000 Spectrophotometer (Thermo Scientific, Wilmington, DE, USA), standardized to 200 ng/µL, and then stored at −20°C. A TaqMan probe based Quantitative real-time PCR (qPCR) assay was used to determine the prevalence and intensity of *P. marinus* and *H. nelsoni.* qPCR reactions were run on Applied Biosystems StepOnePlus real-time PCR machine using the primers, TaqMan probes, and protocols for *P. marinus* (ITS1 gene; Gauthier et al. 2006) and for *H. nelsoni* (18S rDNA gene; Wilbur et al. 2012). To generate standard curves for assessment of parasite intensities, laboratory-synthesized double-stranded DNA fragments overlapping the amplicons for *P. marinus* and *H. nelsoni* (Eurofins Genomics, Louisville, KY, USA) were used. Briefly, we made seven 10-fold dilutions ranging from 10 to 10 000 000 gene copies using stock solution to construct standard curves. Parasite DNA standards and negative controls (RNA-se free water) were run in triplicate. To screen for *P. marinus*, each 15 µL reaction contained 1 µL template DNA, 3.6 µL RNase-free water, 7.5 µL Taqman® Master Mix, 1.2 µL forward primer, 1.2 µL reverse primer and 0.5 µL probe (2.9 µL primer-probe mater mix). To screen for *H. nelsoni,* each 15 µL reaction contained 1 µL template DNA, 2.1 µL RNA-se free water, 7.5 µL Taqman® Master Mix, 1.2 µL forward primer, 1.2 µL reverse primer and 2 µL probe (4.4 µL primer-probe master mix).

StepOnePlus V2.3 software was used to calculate critical threshold (Ct) and gene copy number values. Results from qPCR indicate the quantity of parasite gene copies per 200 ng of genomic DNA from within each 15 µL reaction. The parasite was considered to be present within the oyster host in any sample in which the parasite DNA was detected. Infection intensity was the abundance of the parasite DNA within the host, which correlated to the measure of gene detection quantity per sample.

### Data analysis

To examine patterns in parasite prevalence, we calculated the prevalence of *P. marinus* and *H. nelsoni* separately as the number of infected oysters divided by the total number of oysters collected during each sampling event at each site. We then calculated mean percent prevalence and intensity for both parasites at each site across the cumulative April through October sampling period. Intensity was defined as described above. To determine if parasite prevalence or intensity differed between sites, a one-way analysis of variance (ANOVA) was performed with the random effect of site and parasite prevalence or intensity as the categorical or numerical response variable, respectively. To assess differences among individual sites, post-hoc multiple comparisons were made using the Tukey’s HSD test. Prior to conducting the one-way ANOVA, data were tested for the assumption of normality using a Shapiro–Wilks test and heteroscedasticity using a Levene’s test. To assess if *P. marinus* and *H. nelsoni* randomly associate with one another across the 4 study sites, co-infection was examined using a Chi Square Test of Independence using the observed and expected number of individuals co-infected by both parasites.

To determine the most influential water quality parameters and times in driving oyster parasite prevalence and intensity, we obtained water quality (temperature, salinity, DO, pH) data, collected every 15 minutes at each of the 4 SWMP stations from the NERR Centralized Data Management Office (CDMO; https://cdmo.baruch.sc.edu/). We calculated the mean, maximum, minimum and variance of temperature, salinity, DO and pH for the month of, 1 month prior to, and 2 months prior to each collection date. We used a Pearson’s correlation matrix to assess collinearity among water quality parameters, and if correlation coefficients were greater than 0.7, they were excluded from being used in the same model.

To prevent model redundancy, we independently ran binomial mixed models with a logit link using individual water quality variables for fixed factors (minimum temperature month of, mean salinity month of, mean temperature 1 month prior, minimum DO 2 months prior, etc.) with the random effect of site. From the best-fit models of water quality variables, the fixed factors were extracted and included in a full model with the top predictor water quality variables combined. We then ran a series of generalized linear models with a logit link to assess the presence (1: present, 0: absent) of *P. marinus* and *H. nelsoni* independently. To assess the intensity (cell detection quantity) of each parasite, we ran a series of generalized linear models without a logit link and a normal distribution on log transformed intensity values [log (parasite cell detection quantities/200 ng template DNA)]. *Perkinsus marinus* and *H. nelsoni* presence and intensity were modeled as a function of temperature, salinity, DO and pH as fixed effects; because there was no replication within sites, site was not included as a random effect in generalized linear models. Models that produced the greatest *R*^2^ values were ranked to determine which combination of environmental factors was most influential in driving *P. marinus* and *H. nelsoni* presence and intensity. All models were run using the lme4 package in R (Bates et al. 2015).

We used the ‘dredge’ function in the MuMIn package (Bartoń, 2015) to compare every possible combination of water quality parameters in top fit models against one another. Akaike information criterion corrected for small sample size (AICc) were examined for each model output, and the difference between AICc for a given model and the model with the minimum AICc (ΔAICc) was calculated. For each response variable, we considered the top model (i.e., lowest AICc), and models with ΔAICc < 2 for interpretation. Statistical analyses were performed in R version 4.4.1 (R Development Core Team, 2025).

## Results

### Overall parasite patterns

A total of 882 oysters, ranging from 11 to 147 mm LVL were harvested during the 7-month sampling period. To explore parasite patterns, 439 of those oysters across all sites and timepoints were subsequently examined, ranging from 11 to 138 mm LVL, and 388 oysters had at least one parasite species present (either *P. marinus* or *H. nelsoni,* or both) for an overall prevalence of 88.4% (Table S1). In addition, 34.4% of all oysters sampled were co-infected by both parasites and there was a higher-than-expected probability of coinfection between these two parasites (Chi-square, *X*^2^ = 158.58, *P* < 0.001). Including co-infected oysters, 45.3% of oysters across all sites and timepoints were infected by *P. marinus* (Dermo) and 77.5% of oysters were infected by *H. nelsoni* (MSX) across all time points.

**Figure 2.**
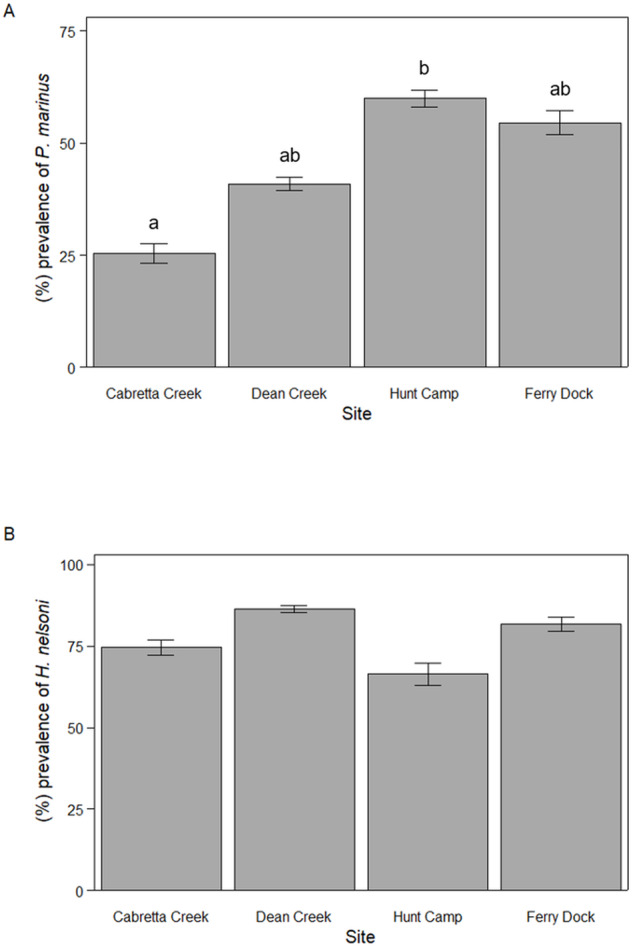
Mean parasite prevalence from April through October 2023 for (A) *P. marinus* and (B) *H. nelsoni* across sites on Sapelo Island, GA, USA. Letters denote statistically significant differences.

For *P. marinus*, mean percent prevalence throughout the April to October sampling period varied among sites with 25.5 ± 6.9% (mean ± SE) of oysters infected at Cabretta Creek, 40.9 ± 11.2% at Dean Creek, 60.0 ± 6.0% at Hunt Camp and 54.6 ± 8.6% at Ferry Dock (ANOVA, *F*_3,38_ = 3.58, *P* = 0.023; Figure 2A). Mean percent prevalence was 34.5% higher at Hunt Camp than Cabretta Creek (Tukey HSD, *P* = 0.027) but was not statistically different across other site combinations. Mean percent prevalence for *H. nelsoni* was less variable and not significantly different among sites; 74.5 ± 7.4% of oysters were infected at Cabretta Creek, 86.4 ± 3.4% at Dean Creek, 66.4 ± 10.6% at Hunt Camp and 81.8 ± 6.9% at Ferry Dock (ANOVA, *F*_2,39_ = 0.816, *P* = 0.493; Figure 2B).

**Figure 3.**
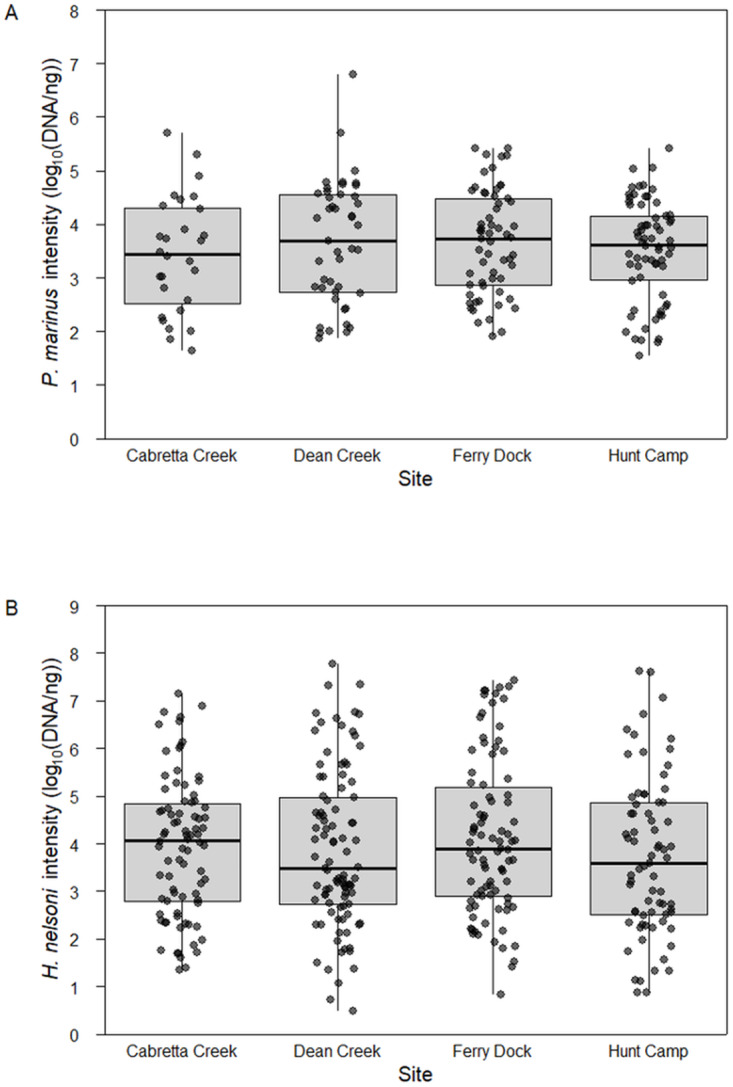
Mean parasite intensity (log scaled) from April through October 2023 for (A) *P. marinus* and (B) *H. nelsoni* across sites on Sapelo Island, GA, USA.

Mean intensity of *P. marinus* was relatively high and did not differ across sites (ANOVA, *F*_3,195_ = 0.499, *P* = 0.683). When *P. marinus* was detected, mean log quantity was 3.31 ± 0.23 DNA copies per 200 ng tissue (1.66 × 10^6^ ± 1.2 × 10^5^ copies per gram tissue; mean ± SE) at Cabretta Creek, 3.65 ± 0.17 at Dean Creek, 3.51 ± 0.11 at Hunt Camp and 3.66 ± 0.12 at Ferry Dock (Figure 3A). Likewise, the mean intensity of *H. nelsoni* was not different across sites (one-way ANOVA, *F*_3,336_ = 0.926, *P* = 0.492). When *H. nelsoni* was detected, mean log quantity was 3.93 ± 0.16 DNA copies per 200 ng tissue at Cabretta Creek, 3.87 ± 0.16 at Dean Creek, 3.71 ± 0.19 at Hunt Camp and 4.12 ± 0.17 at Ferry Dock (Figure 3B).

### Collinearity and reduction of parameters

There was a strong positive correlation between the mean, maximum, and minimum for temperature within the same time-period [e.g., mean temperature 2 months prior and minimum temperature two months prior were highly correlated (*r* = 0.95)]. The following correlation coefficients were greater than 0.7 (or less than − 0.7) and therefore not included in full models: maximum temperature 1 month prior and mean DO 1 month prior (*r* = − .83) and maximum temperature 2 months prior and mean DO 1 month prior (r = − 0.77). Across all models, pH performed poorly as a predictor variable for the prevalence and intensity of both parasites compared to temperature, salinity, DO; therefore, pH was not incorporated into full models.

### Drivers of parasite prevalence and intensity

#### Perkinsus marinus *prevalence*

**Figure 4.**
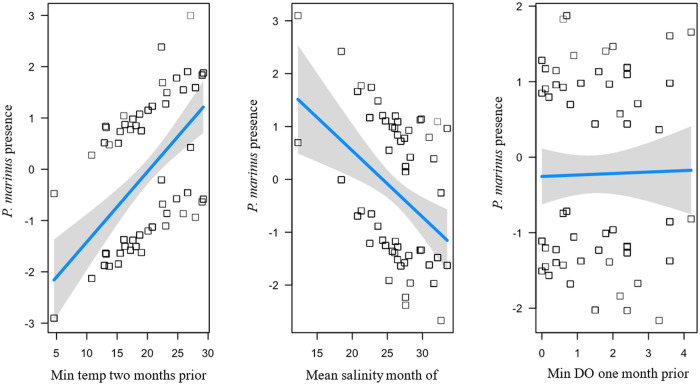
Influence of environmental variables from the model with the lowest AICc and highest *R*^2^ for *P. marinus* presence of individual oysters at each site on Sapelo Island, GA, USA. *Perkinsus marinus* presence is a function of minimum temperature 2 months prior, mean salinity month of, and minimum dissolved oxygen 1 month prior. Squares are partial residuals for each variable that take into account the influence of other fixed variables in the model. Trendlines indicate conditional fit of the model.

There was one top fit model explaining *P. marinus* prevalence across all sites (*R^2^* = 0.207; Table 1) and had main effects that included positive predictors of minimum temperature 2 months prior (*β =* 1.57) and mean salinity month of (*β =* 0.049) and a negative predictor of minimum DO 1 month prior (*β = − 0*.24; Figure 4). *Perkinsus marinus* prevalence changed through time at each site in response to the water quality variables (T, S, DO) indicated by the top model (Supplemental Figure S1A-G).

**Table 1.** Models explaining the influence of water quality variables on 4 oyster response variables from oysters sampled across 4 sites on Sapelo Island, GA, USA. Explanatory variables from models include variation in time and summary statistic data for water temperature (^o^C), salinity (ppt) and dissolved oxygen (DO) (mg/L). The best-fit models (ΔAICc value < 2) are shown for each response variable. The standardized beta coefficients with each independent variable are shown for each model. Degrees of freedom (*df*), log likelihood, AICc, ΔAICc and marginal *R*^2^ (*R*^2^*_m_*) are included for each model

		Water quality parameter and time period										
Response variable	Model rank	Temperature		Salinity		DO		*df*	Log likelihood	AICc	ΔAICc	*R*^2^
*P. marinus* prevalence	1	Min 2 months prior	1.57	Mean month of	0.049	Min one month prior	−0.24	8	−265.9	548.3	0.00	0.207
*P. marinus* intensity	1	Mean 2 months prior	0.65	Mean month of	0.38	Mean month of	0.33	6	−444.9	902.2	0.00	0.055
	2	Mean 2 months prior	0.45			Mean month of	0.17	5	−446.5	903.4	1.17	0.039
	3	Mean 2 months prior	0.70	Mean month of	−0.48	Mean month of	0.32	7	−444.4	903.4	1.18	0.058
*H. nelsoni* prevalence	1	Variance 1 month prior	0.99	Mean two months prior	2.30	Mean one month prior	2.22	8	−208.3	433.0	0.00	0.268
*H. nelsoni* intensity	1	Min 1 month prior	−0.57	Min two months prior	−0.76	Mean two months prior	1.51	9	−870.2	1759.0	0.00	0.275
	2	Min 1 month prior	−0.71	Min two months prior	−0.38	Mean two months prior	1.14	8	−871.7	1759.9	0.98	0.269
	3	Min 1 month prior	−0.74	Min two months prior	−0.46	Mean two months prior	1.09	6	−874.2	1760.8	1.87	0.260

#### Perkinsus marinus *intensity*

Three competing models were found to explain *P. marinus* intensity (Table 1), although the best performing model included main effects of mean temperature 2 months prior (*β =* 0.65), mean DO month of (*β =* 0.33) and mean salinity month of (*β =* 0.38) was a poor fit (*R^2^* = 0.05; Supplemental Figure S2).

#### Haplosporidium nelsoni *prevalence*

**Figure 5.**
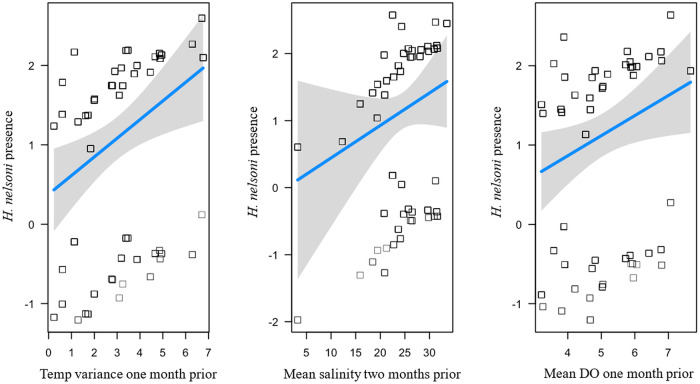
Influence of environmental variables from the model with the lowest AICc and highest *R*^2^ for *H. nelsoni* presence of individual oysters at each site on Sapelo Island, GA, USA. *Haplosporidium nelsoni* presence is a function of temperature variance 1 month prior, mean salinity 2 months prior, and mean dissolved oxygen 1 month prior. Squares are partial residuals for each variable that take into account the influence of other fixed variables in the model. Trendlines indicate conditional fit of the model.

The top fit model explained 26.8% variation of the *H. nelsoni* prevalence (*R^2^* = 0.268; Table 1). The best performing model had main effects of temperature variance 1 month prior (*β =* 2.22), mean salinity 2 months prior (*β =* 2.30) and mean DO 1 month prior (*β =* 0.99; Figure 5). *Haplosporidium nelsoni* prevalence remained high with little variability throughout the sampling period (Supplemental Figure S3A-G).

#### Haplosporidium nelsoni *intensity*

**Figure 6.**
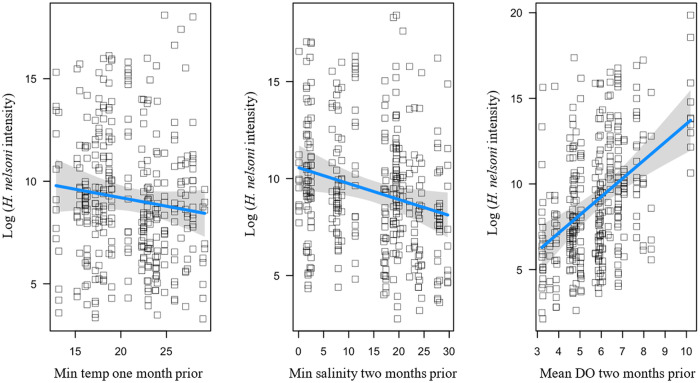
Influence of environmental variables from the model with the lowest AICc and highest *R*^2^ on *H. nelsoni* intensity within individual oysters at each site on Sapelo Island, GA, USA. *Haplosporidium nelsoni* intensity is a function of minimum temperature 1 month prior, minimum salinity 2 months prior, and mean dissolved oxygen 2 months prior. Squares are partial residuals for each variable that take into account the influence of other fixed variables in the model. Trendlines indicate conditional fit of the model.

As with *P. marinus*, three competing models were found to explain *H. nelsoni* intensity (Table 1). The best performing model (*R^2^* = 0.275) had main effects that included a positive predictor of mean DO two months prior (*β =* 1.51) and negative predictors of minimum temperature 1 month prior (*β = − 0*.57), and minimum salinity 2 months prior (*β = − 0*.76; Figure 6).

## Discussion

The protozoan oyster parasites *Perkinsus marinus* and *Haplosporidium nelsoni* exhibit high prevalence and intensity across four sites in Sapelo Island, Georgia – almost 90% of oysters sampled had at least 1 parasite. High prevalence and intensity of these parasites was expected, as the prevalence of parasites from our earlier surveys on Sapelo Island was 93% in the summer (Batchelor et al. 2023). High overall prevalence and/or intensity of both parasites is common throughout southeastern US estuaries in summer surveys (Bobo et al. 1997; Wilbur et al. 2012; Ziegler et al. 2024). The water quality conditions of southeastern estuaries are amenable to the rapid proliferation of both protozoan parasites – when temperatures exceed 20^o^C and salinities are greater than 15 ppt (Ford and Haskin, 1982; Guo and Ford, 2016) – with low summer DO conditions which can impair oyster immune responses (Breitburg et al. 2015). Additionally, oysters in the southeast are almost exclusively intertidal (Bahr and Lanier, 1981), which could also lead to higher parasite prevalence (Malek and Byers, 2017). Thus, the overall conditions common in coastal Georgia and throughout the southeastern USA can cause physiological stress to oysters that maximizes parasite transmission and proliferation (Lafferty and Kuris, 1999; Byers, 2021; Ziegler et al. 2024). The synergistic effects of these environmental factors likely contribute to the complex parasite dynamics in oysters in coastal Georgia.

Both disease-causing parasites showed at least some spatial variability across study sites, despite their relatively close geographic proximity. Natural and anthropogenically driven spatial variation in environmental parameters can result in spatial variation in parasites by directly affecting the host, the pathogen, or both (Breitburg et al. 2015). At large scales, increased heterogeneity in physical and biological conditions can lead to high variability of parasites (Byers et al. 2008). In oysters, recent studies have shown that parasite prevalence is universally high across a relatively large spatial scale (e.g., the Georgia coast; Batchelor et al. 2023) and driven by a combination of both local and landscape-level factors (Ziegler et al. 2024). However, these previous studies have sampled at just one or a few timepoints when prevalence is expected to be highest, so universally high prevalence across large spatial scales may be an artefact of sampling. Unlike many previous studies, we sampled biweekly across an entire season, which was critical to demonstrate fine-scale spatiotemporal variability in parasite prevalence and link the parasites to multiple environmental factors. While temperature and salinity exceeded critical parasite thresholds across all sites and sampling timepoints, there was variability in water quality conditions among the study sites which likely influenced our observed parasite variability.

For *P. marinus,* both temperature and DO were important predictor variables in the best fit model. This parasite tends to proliferate once water temperatures exceed 20^o^C (Guo and Ford, 2016), and higher temperatures lead to increased parasite metabolism and replication within hosts, thereby accentuating parasite transmission and spread (Harvell et al. 1999; Lafferty, 2009; Byers, 2021). Additionally, as monthly temperatures increase throughout the year and affect covarying factors such as DO, it may lead to oyster physiological stress and compromise their ability to fight infection, thus allowing parasite abundance to increase within oyster hosts and spread throughout the water column at key times during the year (Ewart and Ford, 1993; Cook et al. 1998; Breitburg et al. 2015; Malek and Byers, 2017). Thus, we observed prevalence at Cabretta Creek, Dean Creek and Ferry Dock increase throughout the sampling period, peaking in the late summer to early fall when temperature was highest and DO was lowest, while we observed high prevalence with low variability at Hunt Camp. Salinity was also included in the top models for *P. marinus* prevalence and intensity; however, it unexpectedly was negatively correlated with both response variables. Literature suggests that *P. marinus* also increases with salinity (Paynter and Burreson, 1991; Burreson and Ragone Calvo, 1996), but the overall high (euryhaline) salinity conditions with low variability across sites during the sampling period may explain the model results. Alternatively, perhaps this parasite prefers intermediate salinity causing proliferation and abundance to decrease as waters become more oceanic (Batchelor et al. 2023).

When exploring environmental drivers of *P. marinus*, there also appears to be a time-delay in the relationship between water quality parameters and increased parasite prevalence.

Lag-effects of environmental parameters on parasites occur in several other host–parasite interactions. For example, in temperate boreal forests, rainy summers can lead to high prevalence and intensity of the nematode *Howardula aoronymphium* in mycophagous *Drosophila* in the fall and the following spring (Jaenike, 2002). In the U.S. Pacific northwest, experimental studies and field observations examining environmental triggers for outbreaks of sea star wasting disease (SSWD) found that SSWD pathology is temperature dependent and mass mortalities of sea stars tend to occur shortly after temperature increases (Dungan et al. 1982; Bates et al. 2009). Collectively, these studies demonstrate that environmental variables can have delayed impacts on marine parasitism and disease prevalence. Understanding these time-lagged relationships is crucial for predicting and managing disease outbreaks in marine ecosystems.

Unlike *P. marinus,* the prevalence of *H. nelsoni* was high across all sites and less variable throughout the year. Previous studies suggest that *H. nelsoni* is more likely to infect juvenile oysters (Ford et al. 2018). However, we observed high prevalence across all size classes, indicating that the parasite can persist in oysters as they grow into larger size classes (Ford and Haskin, 1982; Ford et al. 2018). Temperature, salinity and DO were all strong predictor variables in the top model and positively correlated with *H. nelsoni* prevalence, but specifically, the best model suggested that as variance in environmental conditions (i.e., temperature) increased, parasite prevalence increased. Batchelor et al. (2023) found that sites with the highest variance in salinity exhibited the highest prevalence and intensity of *H. nelsoni*, suggesting infections may be more common in locations with more variable water quality conditions that could be physiologically stressful for oysters and increase their susceptibility to parasitism (Lafferty and Kuris, 1999; Batchelor et al. 2023). A study by Paull et al. (2015) found that the amplitude and frequency of temperature variability impacted the immune system and energy reserves of the freshwater snail host *Helisoma trivolvis,* thereby increasing the proliferation and transmission of its trematode parasite *Ribeiroia ondatrae*. Additionally, short-term temperature fluctuations increased the prevalence and intensity of *Ordospora colligata* infections in *Daphnia magna*, due to increased host physiological stress and impaired immune defenses under variable environmental conditions (Krichel et al. 2023). The aforementioned studies underscore the importance of considering not just average environmental conditions, but also fluctuations and extremes, when predicting parasite dynamics in changing ecosystems. Alternatively, the lifecycle of *H. nelsoni* may involve an unidentified intermediate host (Ford et al. 2018), so the most influential environmental factors associated with *H. nelsoni* infection in oysters may instead be those that are most suitable for the intermediate host. Future research should focus on understanding the complex life cycle of *H. nelsoni* as well as the progression of disease in both juvenile and adult oysters.

One important caveat to these results is that the models in the present study have limited predictive power. Each top fit model explained less than 30% variation in *P. marinus* and *H. nelsoni* prevalence and intensity, which suggests that environmental or other factors not included in the analyses of the present study may be more influential in driving the intensity of both parasites within oyster hosts. Water flow can be an important determinant in parasitic infections (Lafferty and Kuris, 1999) but was not measured in the current study. Likewise, geographic features at the seascape scale can also influence parasite dynamics in coastal systems. For example, sites with greater marsh area around a reef relative to open water reduced *P. marinus* prevalence and intensity but increased *H. nelsoni* prevalence (Ziegler et al. 2024), while *P. marinus* may increase with distance from freshwater (Hanley et al. 2019). Parasite prevalence and intensity can also respond to multi-year precipitation cycles and the El-Nino Southern Oscillation (Powell et al. 1992; Soniat et al. 2012). In this study, the prevalence of *P. marinus* was ∼40% lower on average than a survey at the same sites in 2018, while the prevalence of *H. nelsoni* in this study was ∼18% higher than 2018 (Batchelor et al. 2023), suggesting that the factors affecting annual or multiannual variability could mask within-year or within-season trends. Finally, the health of the host is also critical for parasite infection and intensity, since physiological stress dampens host ability to fight infection and has been shown to correspond to higher level of parasitism (Oppliger et al. 1998; Lafferty and Kuris, 1999; Malek and Byers, 2017). All oysters in this study come from intertidal populations, which tend to have more intense infections than subtidal populations (Malek and Breitburg, 2016; Malek and Byers, 2017), likely due to the more extreme and stressful physiological conditions of repeated daily tidal exposure. Sampling intertidal oysters could be masking the effects of water quality parameters in driving *P. marinus* and *H. nelsoni* intensity (Oppliger et al. 1998; Lafferty and Kuris, 1999; Malek and Byers, 2017).

The parasites examined in this study – *P. marinus* and *H. nelsoni –* typically lead to oyster diseases, Dermo and MSX, respectively, that have negative ecosystem-level and economic consequences (Mackin et al. 1950; Ford and Haskin, 1982; Andrews, 1988; Burreson and Ragone Calvo, 1996) by reducing oyster health, reproductive output and even leading to mortality events (Burreson and Ragone Calvo, 1996). Exploring the individual- and ecosystem-level consequences of parasite infection was beyond the scope of this study, but the parasites do not appear to have negative consequences locally. Despite high parasite prevalence and intensity on Sapelo Island during the sampling period of this study, oyster populations appeared to be in good condition (high oyster density and reef complexity; *personal observation*). In this study, only a few oysters had no detectable parasite loads, and parasite intensity was highly variable among individual oysters collected from within a single site. Thus, it is possible that we sampled oysters with parasite loads that were not high enough to cause adverse health effects or mortality simply because those oysters were already deceased at the time of collection. Alternatively, we used qPCR amplification of parasite DNA to detect prevalence and intensity, which may be a more sensitive and accurate approach but cannot determine the parasite life or infective stage in the host (Gauthier et al. 2006; Wilbur et al. 2012). Though qPCR is a highly sensitive detection method and can explain findings for *P. marinus* and *H. nelsoni* in this study, cases with low intensity may involve underdeveloped parasites that do not affect the oyster, and high prevalence may be common, but these infections may be asymptomatic (Batchelor et al. 2023).

However, our observation of limited negative impacts of disease-causing protozoan parasites on oyster populations in this study aligns with several recent studies in southeastern U.S. estuaries. Recent studies in coastal Georgia that found that the integrity of reef structure remained intact despite universally high parasite prevalence across a large spatial scale (150 km) and a lack of negative impacts for *P. marinus* and *H. nelsoni* on oyster condition index (Batchelor et al. 2023; Ziegler et al. 2024). In Florida, oysters with *P. marinus* may exhibit increased tissue condition relative to oysters without the parasite, although that may have been confounded by tidal elevation (Hanley et al. 2019). Likewise, high protozoan parasite loads are common in North and South Carolina within otherwise healthy oyster populations (Bobo et al. 1997; Wilbur et al. 2012). Intertidal oysters in the southeast USA appear to be resistant or more tolerant to these protozoan parasites (Powell et al. 2011) and/or are more resilient to other stressors that usually co-occur with parasites that lead to disease effects and mortality (e.g., temperature; Chu and Volety, 1997; Malek and Byers, 2018). Alternatively, parasite strains present in this region of the southeast may be less virulent or lethal phenotypes (Bushek and Allen, 1996; Carnegie et al. 2021). It is also possible that high oyster recruitment and growth along the Georgia coast could mask the negative impacts of oysters lost to parasite-related mortality (O’Beirn et al. 1996; Malek and Byers, 2017), although other southeast US estuaries with much lower recruitment (Byers et al. 2015) also exhibit high prevalence of parasites with minimal effects (Wilbur et al. 2012). Nevertheless, from April through October in Georgia, water quality conditions are conducive for both protozoan parasites with seemingly limited impacts on local oyster populations.

Seasonal cycles and tidal influences could also contribute to the apparent tolerance in southeastern oyster populations. Geographic variation of *P. marinus* and *H. nelsoni* infections can in part be attributed to the magnitude and duration of seasonal changes that occur with changes in latitude, tidal emersion and coastal morphologies (Bahr and Lanier, 1981; Ewart and Ford, 1993; Burreson and Ragone Calvo, 1996; Cook et al. 1998; Lenihan et al. 1999; Walles et al. 2016). Historically, *P. marinus* and *H. nelsoni* have had much more of a significant and chronic effect on both wild and cultured oyster populations in the northeastern and mid-Atlantic regions of the USA (especially Chesapeake and Delaware bays; Ewart and Ford, 1993; Burreson and Ragone Calvo, 1996; Cook et al. 1998), and are often linked to additional stressors, such as high temperatures (Chu and La Peyre, 1993; Ford and Tripp, 1996). In southeastern US estuaries, which tend to be more saline and warmer even in the winter, oysters are exposed to potential parasite infections year-round (Andrews, 1988; O’Beirn et al. 1994; Oliver et al. 1998), whereas oysters in mid-Atlantic and northeastern US estuaries experience temperature and salinity regimes in winter months that reduce parasite exposure (Andrews, 1988; Carnegie and Burreson 2012; Oliver et al. 1998). Additionally, the distribution of oysters throughout much of the southeastern USA is almost exclusively intertidal (Bahr and Lanier, 1981; Walles et al. 2016; Malek and Byers, 2017; Dieudonne and Carroll, 2022). The prevalence and intensity of *P. marinus* and *H. nelsoni* in oysters is highest in the intertidal throughout this entire range (Karolus et al. 2000; Johnson and Smee, 2014; Malek and Byers, 2017), and the changing profile of the intercontinental shelf along the east coast strongly influences tides, waves and flow regimes; subsequently influencing the exposure of oysters to parasites in the water column (Redfield AC, 1958; Bahr and Lanier, 1981; Lenihan et al. 1999). Therefore, the high tidal amplitude, strong tidal forces, exclusively intertidal oyster reefs and environmental conditions that permit *P. marinus* and *H. nelsoni* to survive year-round have most likely led to high, chronic exposure for oysters in this region which may subsequently have led to their potential tolerance to these parasites. Future work should explore additional biological and physical factors influencing the prevalence and intensity of *P. marinus* and *H. nelsoni* as well as the mechanisms for the apparent tolerance of southeastern oyster populations.

In conclusion, our results indicate that *P. marinus* and *H. nelsoni* are ubiquitous across a small spatial scale and over time, and patterns of parasite prevalence and intensity were driven by a combination of water quality conditions. There was a time-lag effect for environmental parameters driving *P. marinus* prevalence that coincided with when water quality conditions were likely most stressful for oysters and/or favorable for *P. marinus* proliferation and transmission (Cook et al. 1998; Breitburg et al. 2015; Malek and Byers, 2017). This same time-lag effect was not seen for *H. nelsoni*; instead, prevalence remained high throughout the year with little variability between sites. Intensity was highly variable among individual oysters from within sites, which could be the result of several other physical factors not investigated in this study or the complex pathophysiology between oyster host innate immune response and the ability of parasites to evade and suppress host defenses (Lenihan et al. 1999; Hughes et al. 2010; Malek and Byers, 2017; Ziegler et al. 2024). Even though this study presented disease data biweekly over a 7-month period, there remains a multitude of potential factors that vary across spatial scales and years that may be driving the complex relationship between oysters and parasites, emphasizing the need to build long-term disease datasets (Carnegie et al. 2021; Batchelor et al. 2023). Changing climatic conditions may also permit parasites to remain sequestered within oysters during the winter period and increase in abundance once environmental conditions are favorable the following spring. Paull et al. (2015) conducted a mesocosm study with the snail host *Helisoma trivolvis* and the virulent parasite *Ribeiroia ondatrae* from September to July and found that warming of 3°C caused snails to release parasites 9 months earlier and increased infected snail mortality by 4-fold. Considering continued climatic changes, expanding parasite ranges, and protracting seasonality of parasites, it becomes paramount for research to continue investigating the influence of temperature, salinity, DO and pH on oyster parasite dynamics across various spatiotemporal scales (Harvell et al. 1999; Lafferty, 2009; Byers, 2021). Understanding this complex environment–host–parasite interaction will not only have implications for management, restoration and aquaculture in regions with high parasite-related mortalities, but also for how parasite prevalence and intensity continue to shape estuarine communities and the services provided by an irreplaceable ecosystem engineer.

## Supporting information

10.1017/S0031182025101431.sm001 Atencio et al. supplementary material Atencio et al. supplementary material

## Data Availability

Data for this work are available upon request.

## Supplementary material

The supplementary material for this article can be found at https://doi.org/10.1017/S0031182025101431.
