# Supplementary material for: Drivers of Perkinsus marinus and Haplosporidium nelsoni prevalence and intensity in oyster reefs around Sapelo Island, Georgia
Source: Parasitology. 2025 Dec 19;153(2):186–97. doi: 10.1017/S0031182025101431 (PMC13215739; doi:10.1017/S0031182025101431)
Supplement: Atencio et al. supplementary material [file S0031182025101431sup001.docx]

**Supplementary materials**

Drivers of *Perkensis marinus* and *Haplosporidium nelsoni* prevalence and intensity in oyster reefs around Sapelo Island, Georgia

Wil E. Atencio^1,2^, Shelby L. Ziegler^3,4^, Stephen E. Greiman^1^, John M. Carroll^1,*^

^1^Department of Biology, Georgia Southern University, Statesboro, GA, USA

^2^Department of Environmental Sciences, University of Virginia, Charlottesville, VA, USA

^3^Department of Biology, Villanova University, Villanova, PA, USA

^4^Center for Biodiversity and Ecosystem Stewardship, Villanova University, Villanova, PA, USA

***Corresponding author**: [jcarroll@georgiasouthern.edu](mailto:jcarroll@georgiasouthern.edu)

**Table S1:** Total number and size range of oysters collected at each site during each sampling visit and the subsequent number and size ranges of oysters sampled for parasites.

|  |  | Total oysters sampled | | Disease samples | | | |
| --- | --- | --- | --- | --- | --- | --- | --- |
| Date | Site | N | Range (mm) | N | Range (mm) | # with P marinus | # with H nelsoni |
| 4/1/2023 | Hunt Camp | 27 | 38-109 | 10 | 46-109 | 3 | 8 |
|  | Cabretta Creek | 13 | 46-138 | 10 | 46-138 | 4 | 6 |
|  | Ferry Dock | 39 | 34-106 | 10 | 34-106 | 2 | 10 |
|  | Dean Creak | 26 | 29-95 | 10 | 29-72 | 2 | 9 |
| 5/1/2023 | Hunt Camp | 39 | 22-102 | 10 | 35-99 | 8 | 10 |
|  | Cabretta Creek | 37 | 11-147 | 10 | 11-79 | 1 | 7 |
|  | Ferry Dock | 33 | 23-113 | 10 | 31-84 | 2 | 9 |
|  | Dean Creak | 46 | 25-100 | 10 | 25-75 | 1 | 9 |
| 5/17/2023 | Hunt Camp | 32 | 23-102 | 10 | 26-73 | 4 | 8 |
|  | Cabretta Creek | 24 | 28-118 | 10 | 28-118 | 2 | 8 |
|  | Ferry Dock | 23 | 24-115 | 10 | 24-115 | 3 | 7 |
|  | Dean Creak | 30 | 26-98 | 10 | 26-98 | 4 | 8 |
| 6/6/2023 | Hunt Camp | 15 | 24-73 | 10 | 24-73 | 6 | 10 |
|  | Cabretta Creek | 19 | 24-111 | 10 | 24-111 | 2 | 10 |
|  | Ferry Dock | 18 | 30-114 | 10 | 44-114 | 8 | 10 |
|  | Dean Creak | 17 | 41-83 | 10 | 41-83 | 5 | 10 |
| 6/26/2023 | Hunt Camp | 18 | 28-92 | 10 | 35-92 | 3 | 8 |
|  | Cabretta Creek | 16 | 27-103 | 10 | 27-102 | 0 | 8 |
|  | Ferry Dock | 20 | 17-83 | 10 | 17-83 | 2 | 9 |
|  | Dean Creak | 19 | 27-106 | 9 | 38-89 | 4 | 9 |
| 7/13/2023 | Hunt Camp | 20 | 28-92 | 10 | 34-92 | 8 | 10 |
|  | Cabretta Creek | 20 | 29-134 | 10 | 33-134 | 3 | 9 |
|  | Ferry Dock | 17 | 21-100 | 10 | 21-100 | 7 | 10 |
|  | Dean Creak | 17 | 33-115 | 10 | 33-89 | 5 | 9 |
| 7/26/2023 | Hunt Camp | 19 | 26-82 | 10 | 29-82 | 8 | 0 |
|  | Cabretta Creek | 15 | 27-100 | 10 | 27-100 | 3 | 1 |
|  | Ferry Dock | 14 | 36-104 | 10 | 36-104 | 4 | 4 |
|  | Dean Creak | 14 | 35-79 | 10 | 39-70 | 4 | 8 |
| 8/9/2023 | Hunt Camp | 15 | 30-96 | 10 | 39-96 | 5 | 5 |
|  | Cabretta Creek | 14 | 40-130 | 10 | 10-130 | 4 | 8 |
|  | Ferry Dock | 15 | 32-100 | 10 | 32-92 | 7 | 6 |
|  | Dean Creak | 15 | 26-118 | 10 | 26-118 | 3 | 8 |
| 9/1/2023 | Hunt Camp | 16 | 29-88 | 10 | 40-88 | 8 | 1 |
|  | Cabretta Creek | 17 | 23-95 | 10 | 23-78 | 1 | 8 |
|  | Ferry Dock | 15 | 22-132 | 10 | 47-132 | 8 | 5 |
|  | Dean Creak | 15 | 33-104 | 10 | 38-104 | 6 | 7 |
| 9/29/2023 | Hunt Camp | 13 | 26-90 | 10 | 26-90 | 6 | 5 |
|  | Cabretta Creek | 15 | 51-115 | 10 | 51-102 | 0 | 7 |
|  | Ferry Dock | 13 | 30-129 | 10 | 31-129 | 9 | 10 |
|  | Dean Creak | 17 | 30-86 | 10 | 30-86 | 5 | 7 |
| 10/25/2023 | Hunt Camp | 17 | 27-73 | 10 | 27-73 | 7 | 7 |
|  | Cabretta Creek | 11 | 21-105 | 10 | 36-105 | 8 | 10 |
|  | Ferry Dock | 15 | 32-105 | 10 | 40-105 | 8 | 10 |
|  | Dean Creak | 12 | 23-105 | 10 | 40-105 | 6 | 10 |
|  | Sum | 882 | 11-147 | 439 | 11-138 | 199 | 338 |


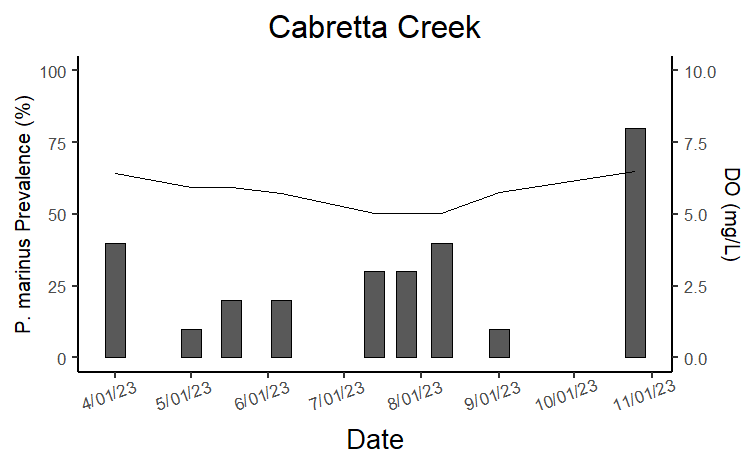


B


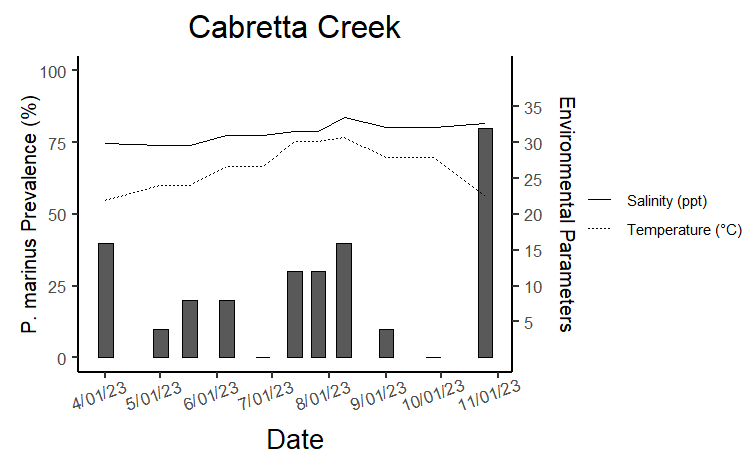


A


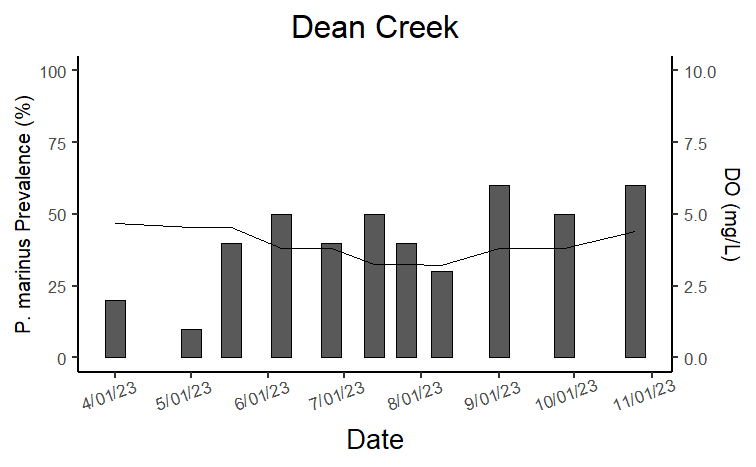


D


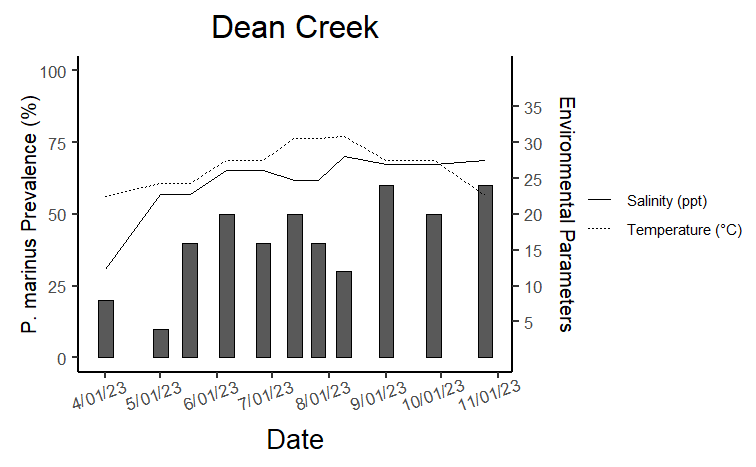


C


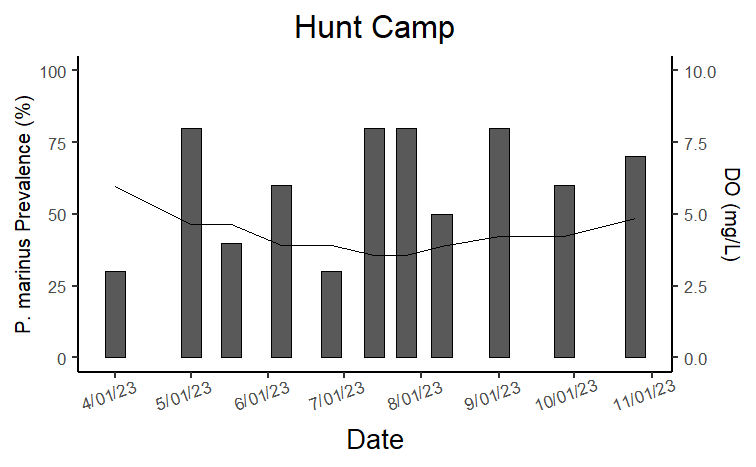


F


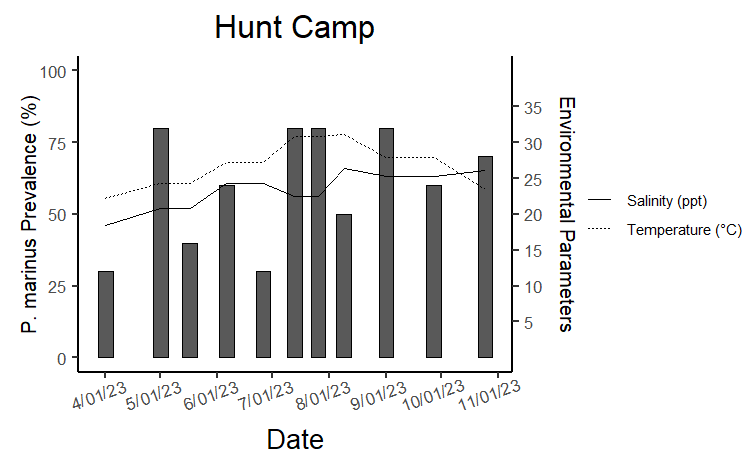


E


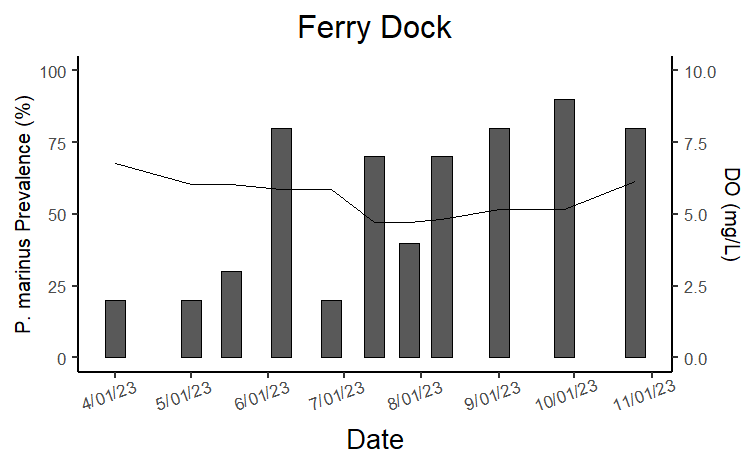


H


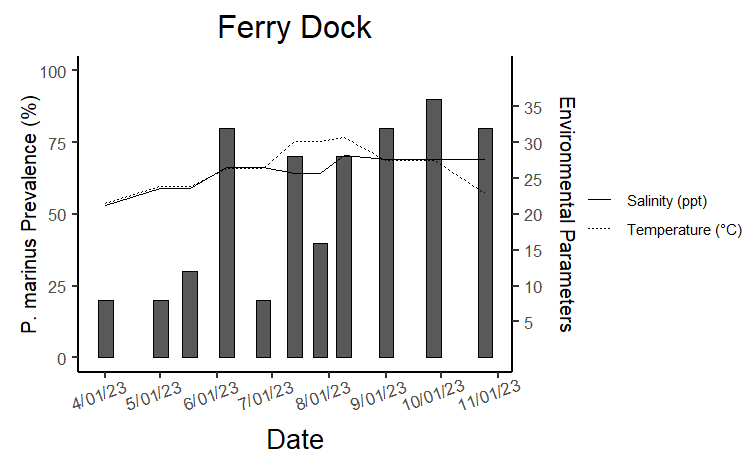


G

**Figure S1:** Overlay plots of most influential predictor water quality variables indicated by top models explaining *P. marinus* prevalence percent at four sites at Sapelo Island, GA. **A, C, E, G** illustrate *P. marinus* percent prevalence in response to temperature (^o^C), and salinity (ppt). **B, D, F, H** illustrate *P. marinus* percent prevalence in response to dissolved oxygen (DO) (mg/L).


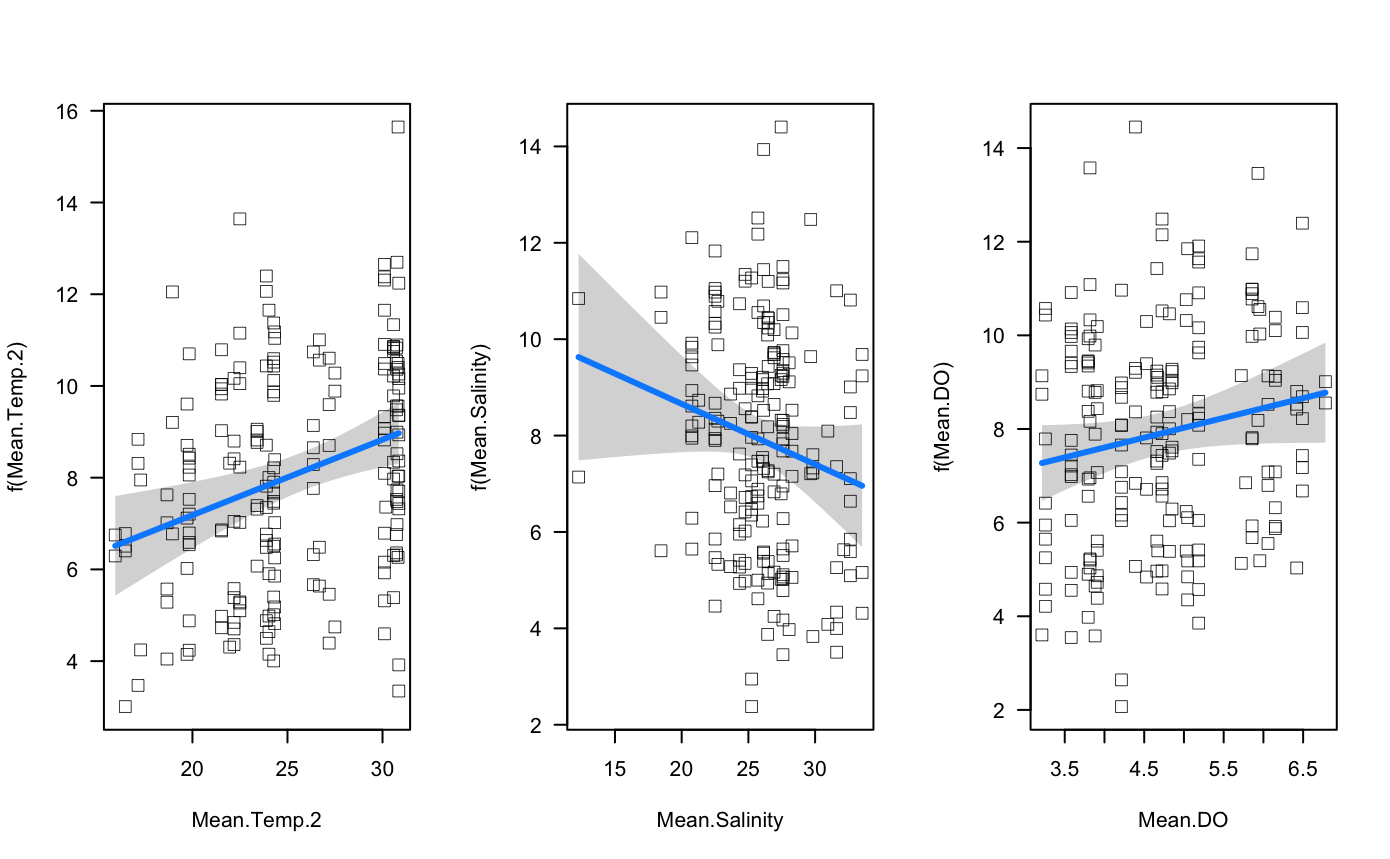


Log (*P. marinus* intensity)

Log (*P. marinus* intensity)

Log (*P. marinus* intensity)

Mean temp two months prior

Mean salinity month of

Mean DO month of

**Figure S2:** Influence of environmental variables from the model with the lowest

AICc and highest *R*^2^ on *P. marinus* intensity within individual oysters at each site on Sapelo Island, GA. *P. marinus* intensity is a function of mean temperature two months prior, mean salinity month of, and mean dissolved oxygen month of. Squares are partial residuals for each variable that take into account the influence of other fixed variables in the model and trendlines indicate conditional fit of the model.


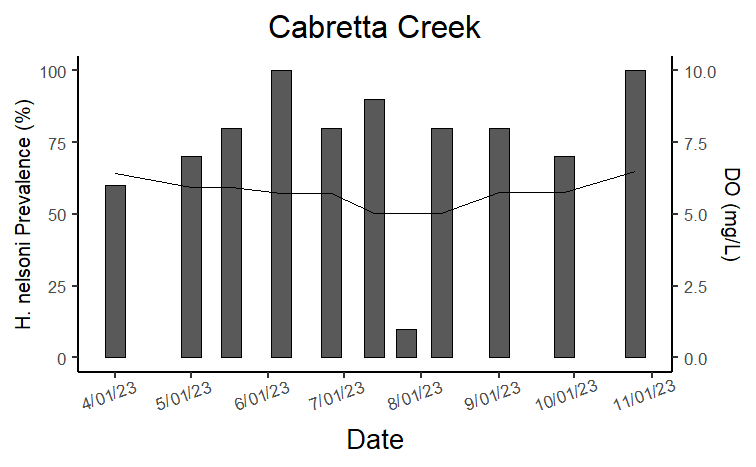


B


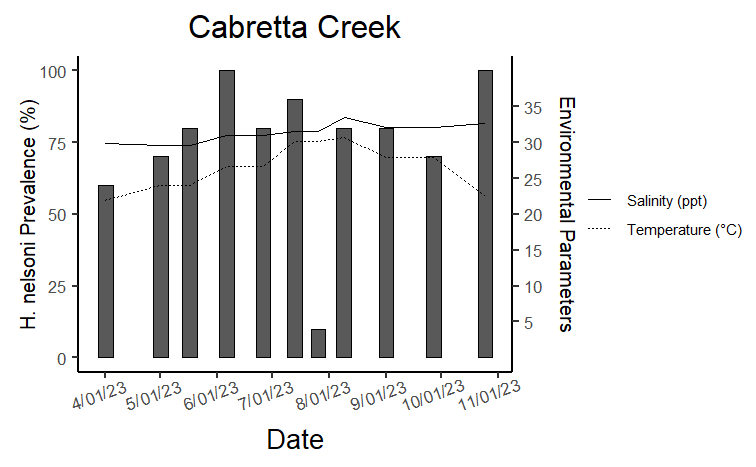


A


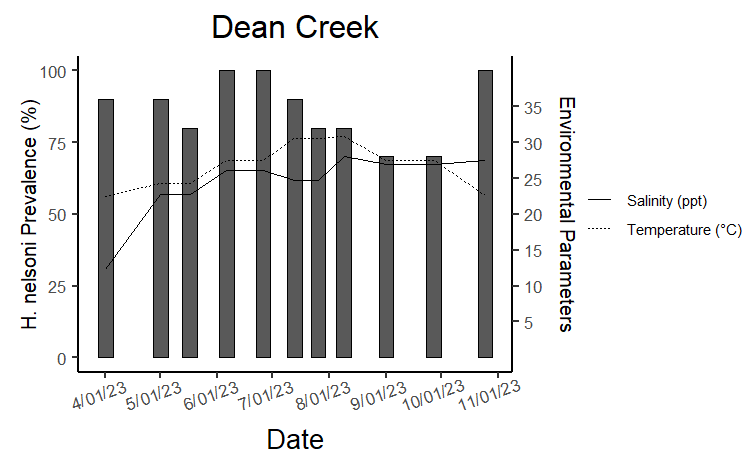


C


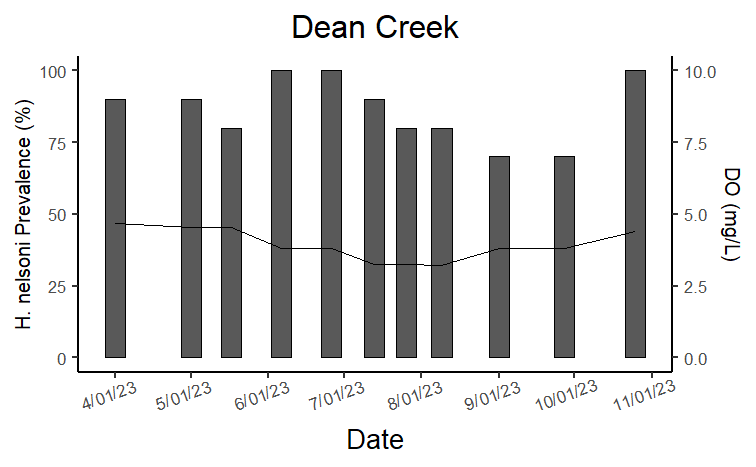


D


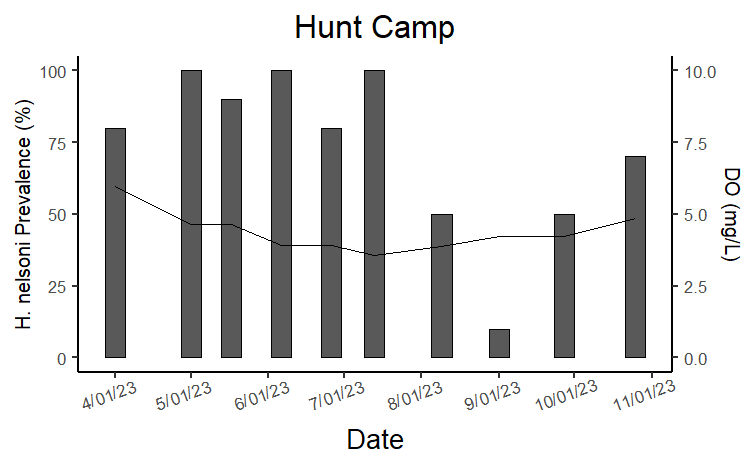


F


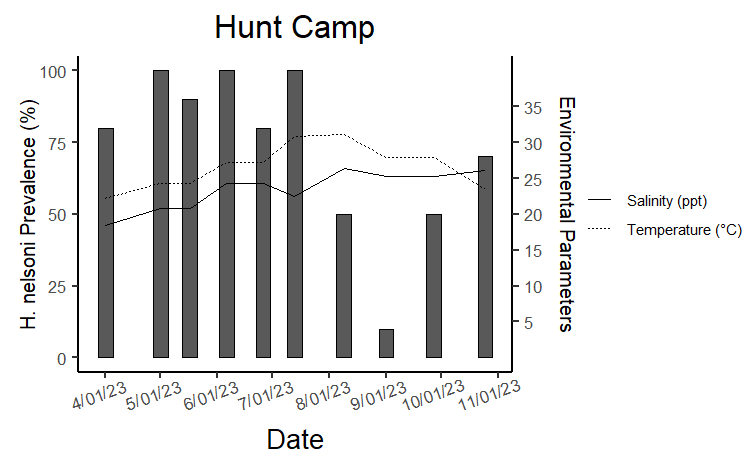


E


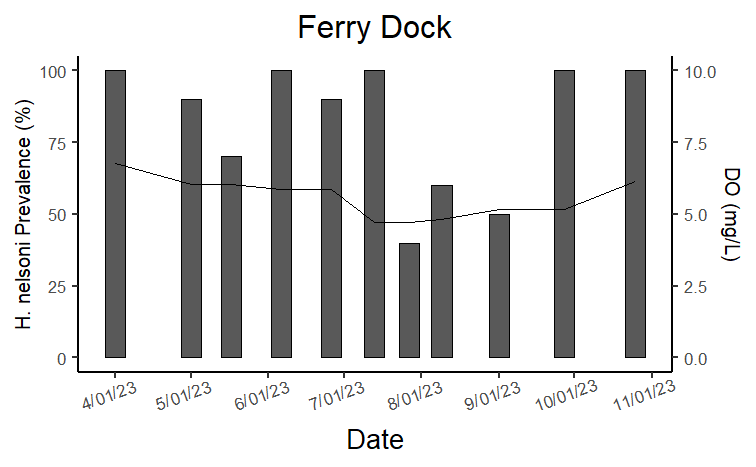


H


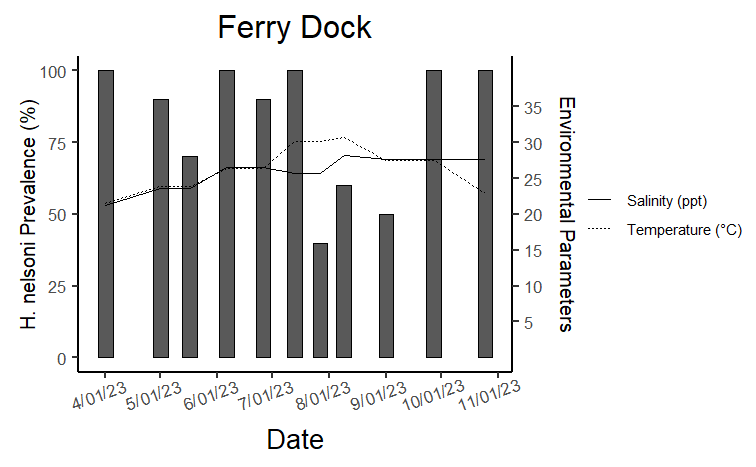


G

**Figure S3:** Overlay plots of most influential predictor water quality variables indicated by top models explaining *H. nelsoni* prevalence percent at four sites at Sapelo Island, GA. **A, C, E, G** illustrate *H. nelsoni* percent prevalence in response to temperature (^o^C), and salinity (ppt). **B, D, F, H** illustrate *H. nelsoni* percent prevalence in response to dissolved oxygen (DO) (mg/L).
